# Supplementary figures and images for: Non-standardized protein background in IVF media linked to serum-derived albumin supplementation
Source: J Assist Reprod Genet. 2025 Sep 13;42(10):3535–49. doi: 10.1007/s10815-025-03616-0 (PMC12602788; doi:10.1007/s10815-025-03616-0)

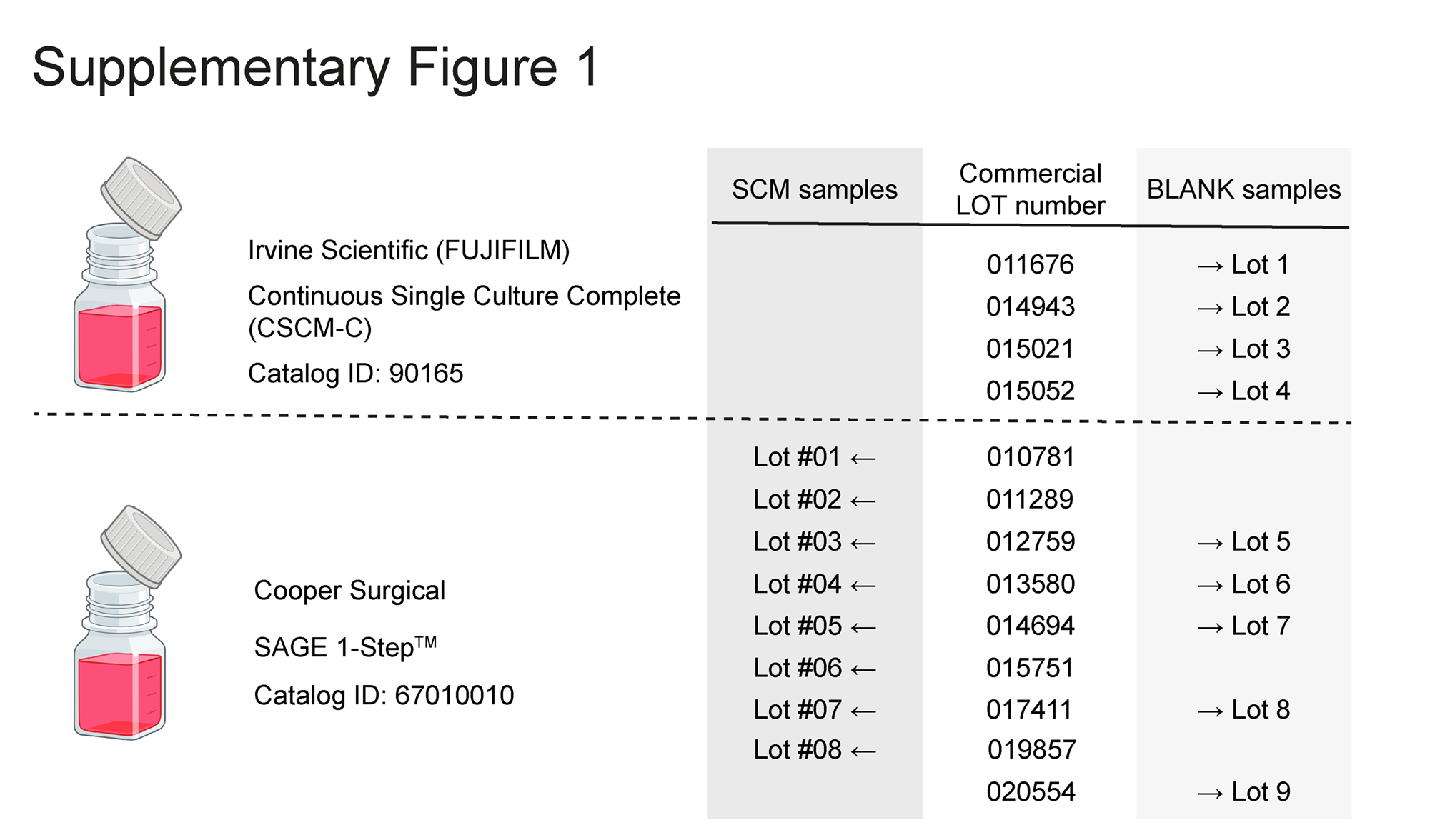

Supplement: Supplementary file 1 — PNG (290 KB) [file 10815_2025_3616_Fig7_ESM.png]

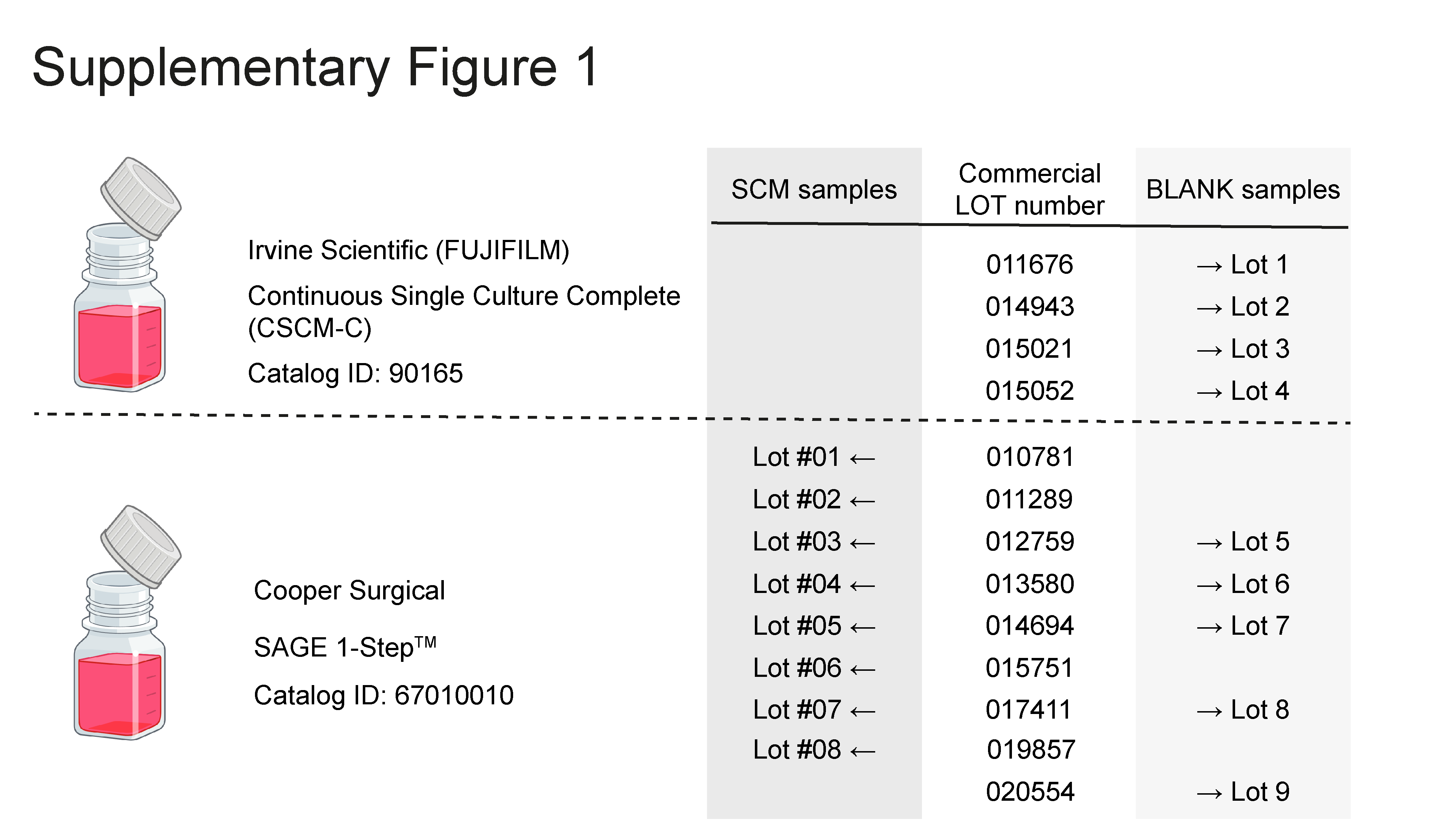

Supplement: Supplementary file 2 — Supplementary file1 (TIF 1529 KB) [file 10815_2025_3616_MOESM1_ESM.tif]

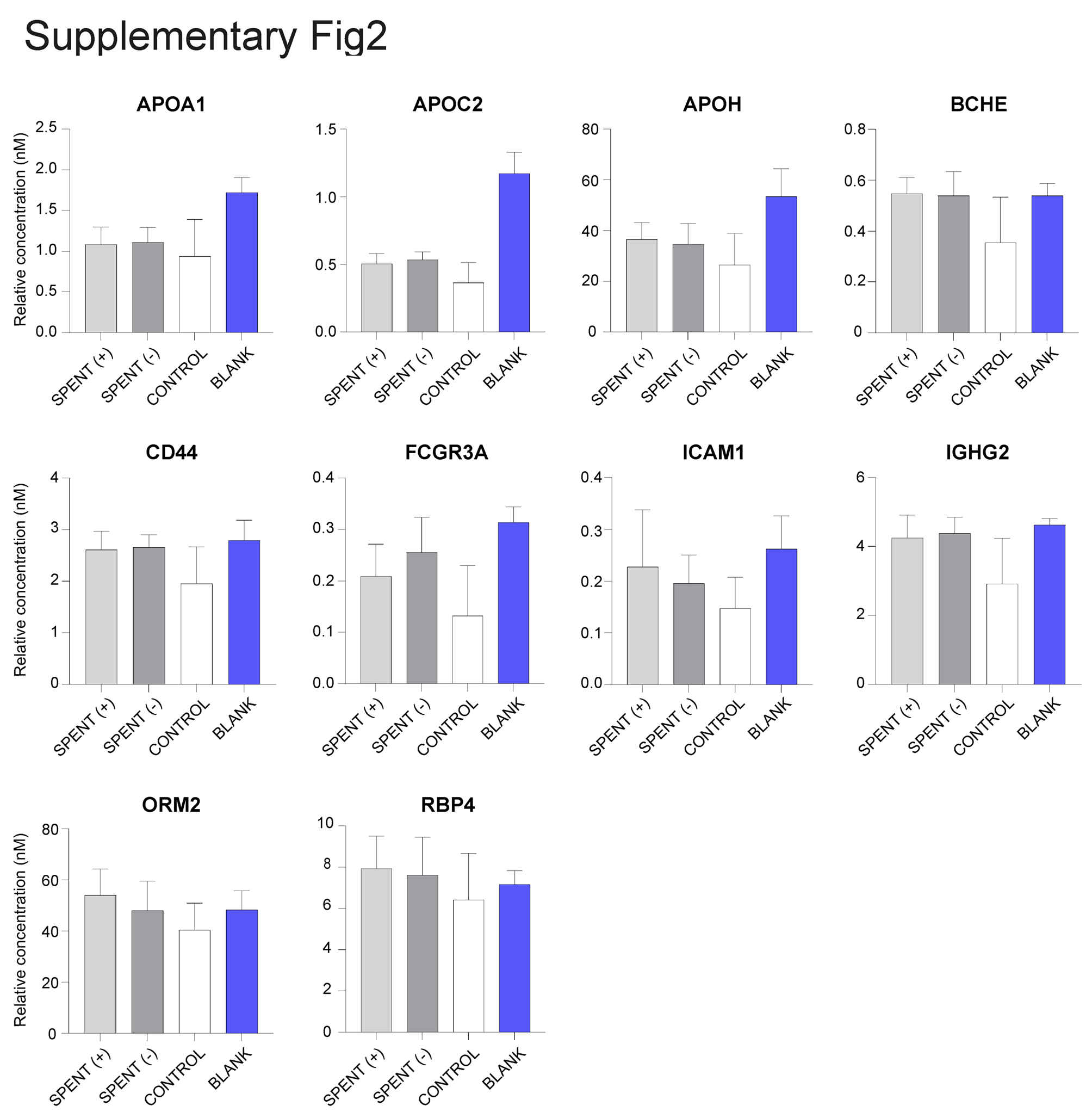

Supplement: Supplementary file 3 — PNG (203 KB) [file 10815_2025_3616_Fig8_ESM.png]

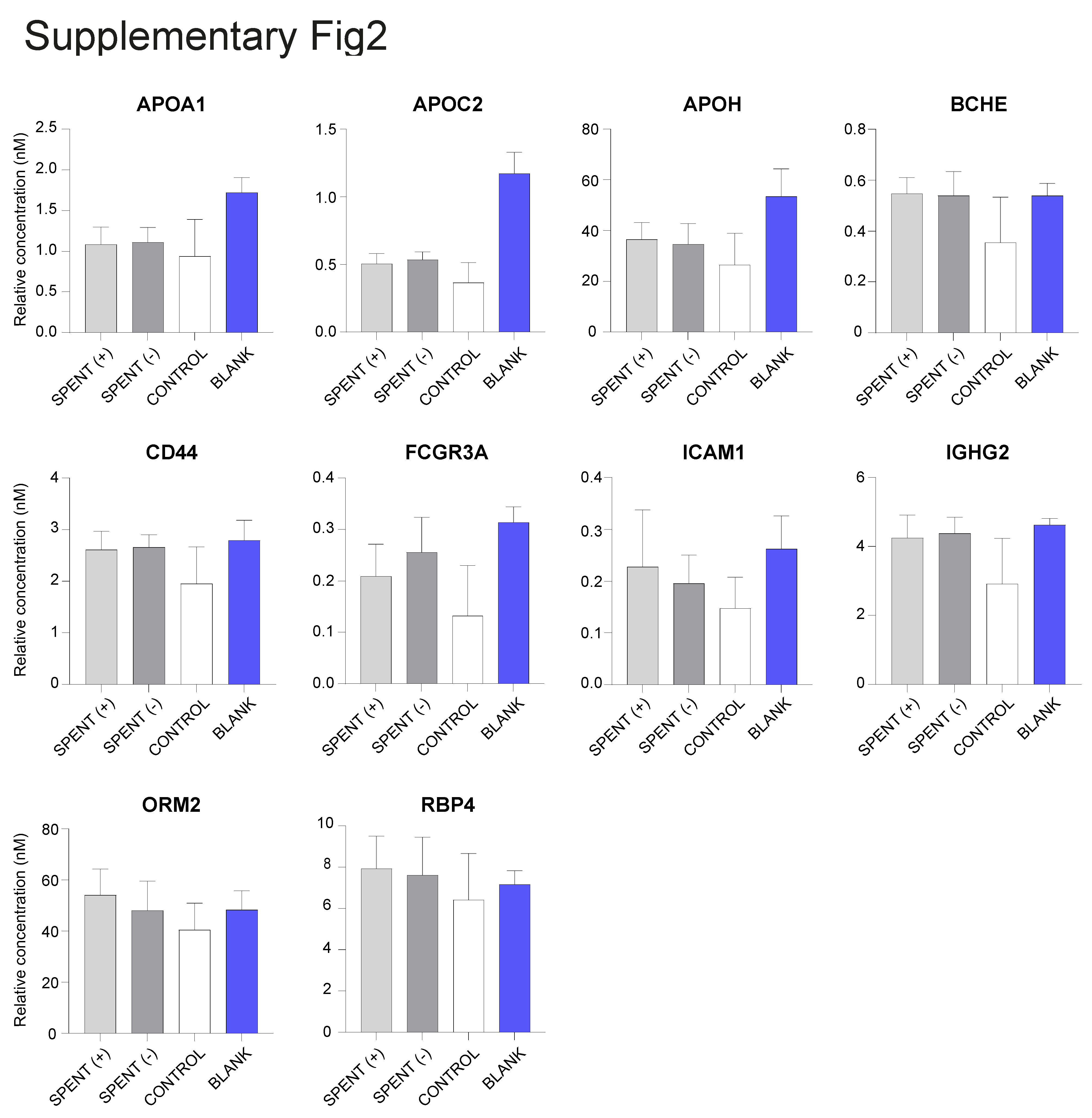

Supplement: Supplementary file 4 — Supplementary file2 (TIF 2419 KB) [file 10815_2025_3616_MOESM2_ESM.tif]

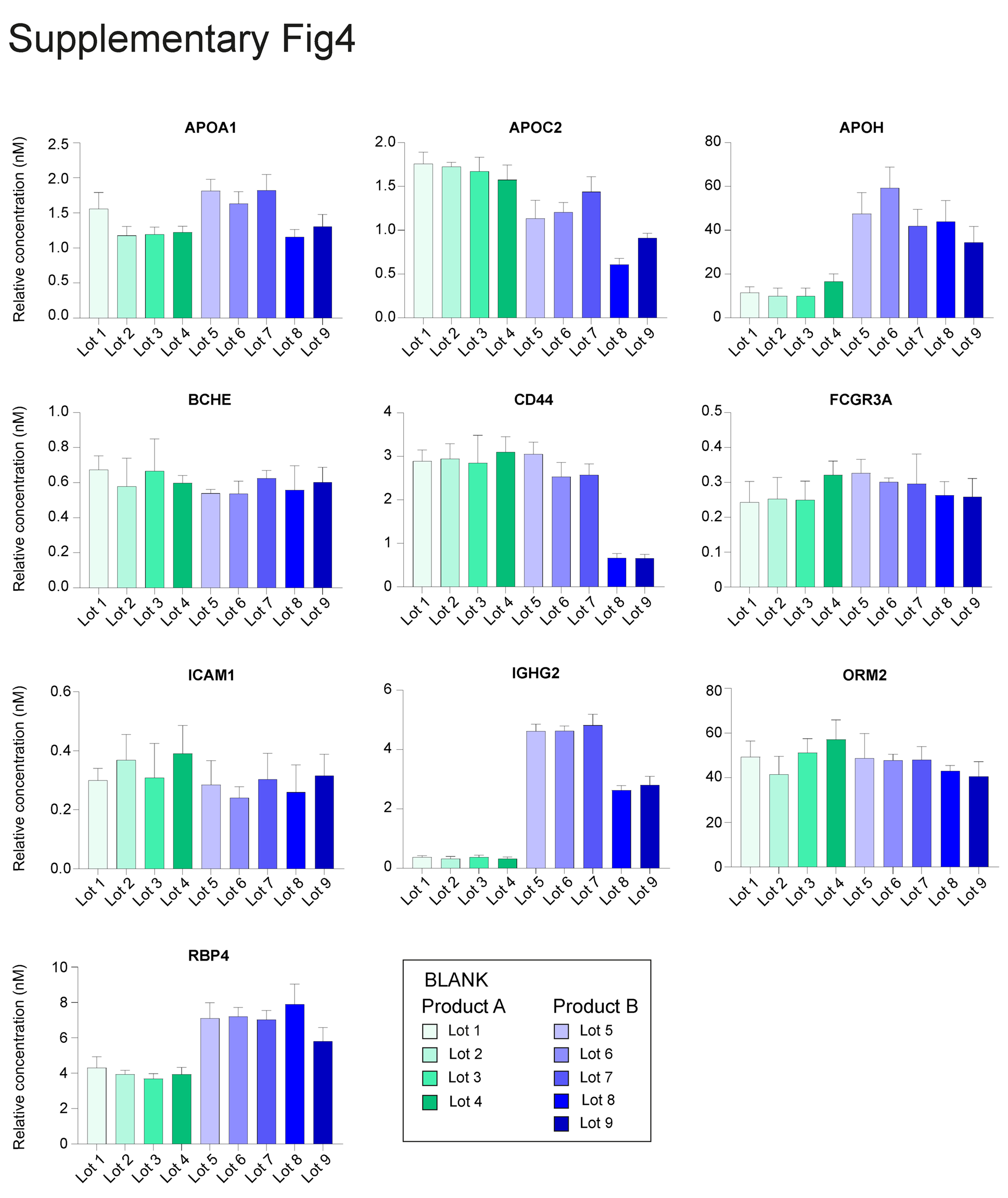

Supplement: Supplementary file 5 — PNG (352 KB) [file 10815_2025_3616_Fig9_ESM.png]

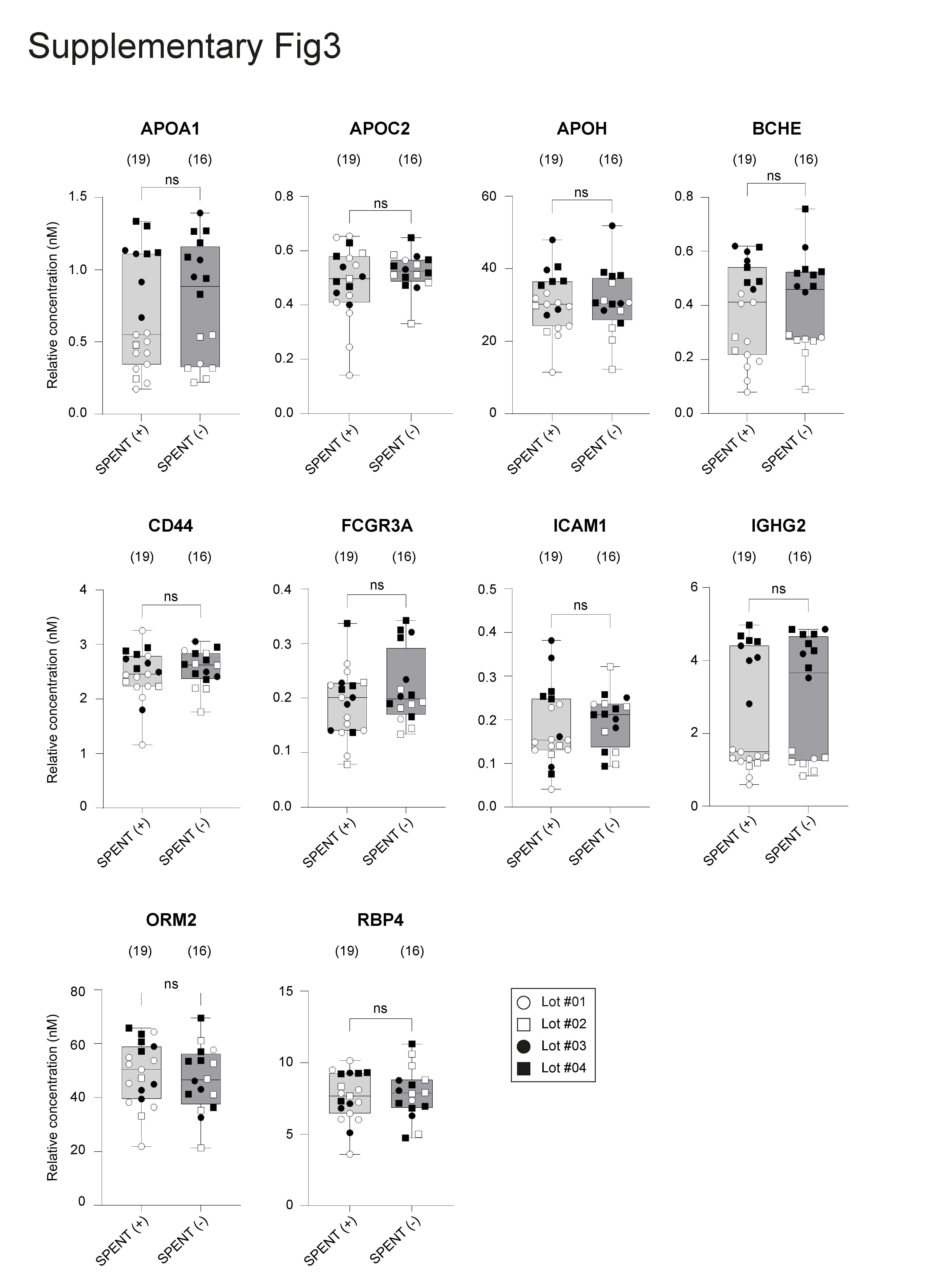

Supplement: Supplementary file 6 — Supplementary file3 (TIF 3038 KB) [file 10815_2025_3616_MOESM3_ESM.tif]

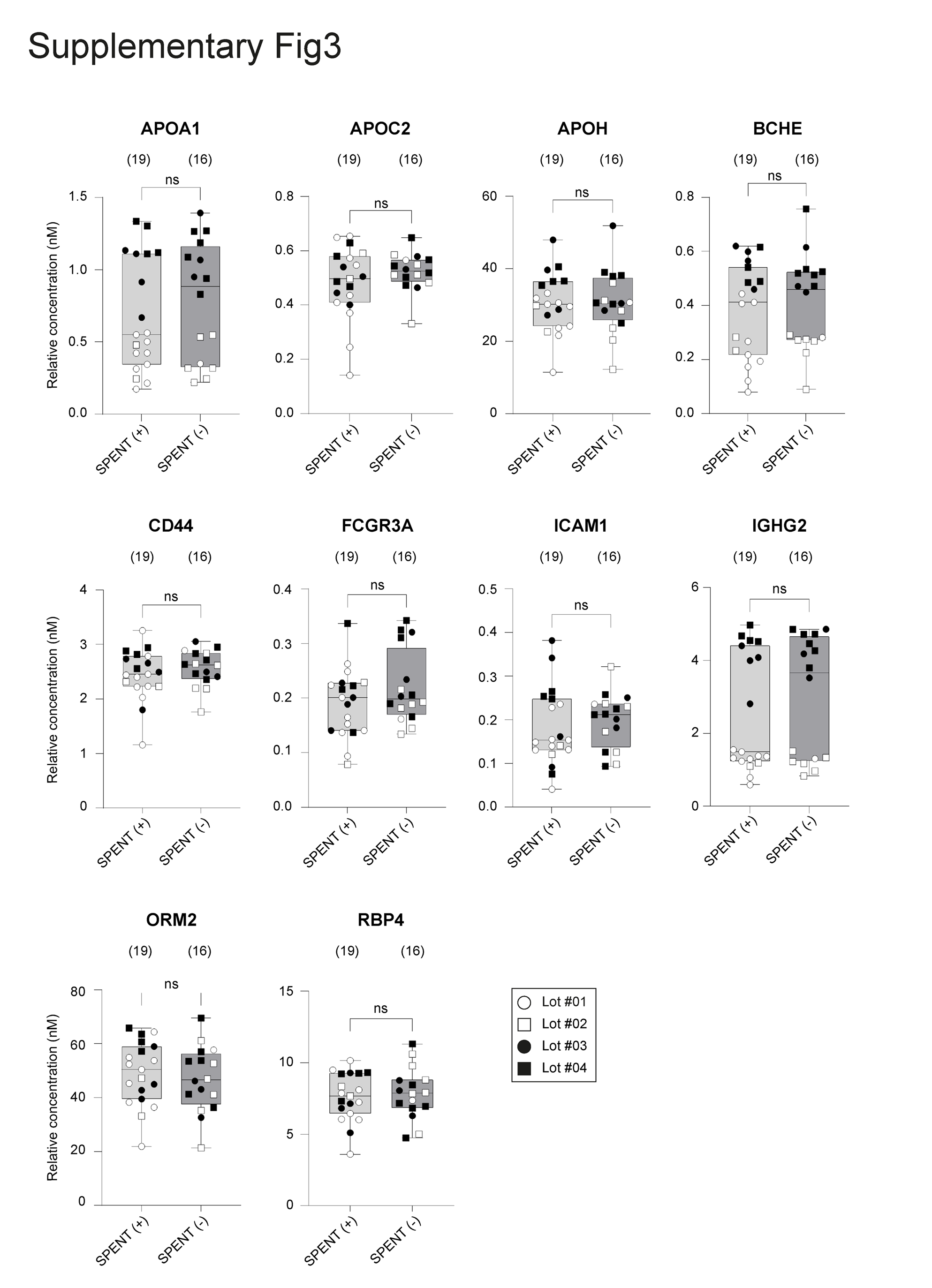

Supplement: Supplementary file 7 — PNG (352 KB) [file 10815_2025_3616_Fig10_ESM.png]

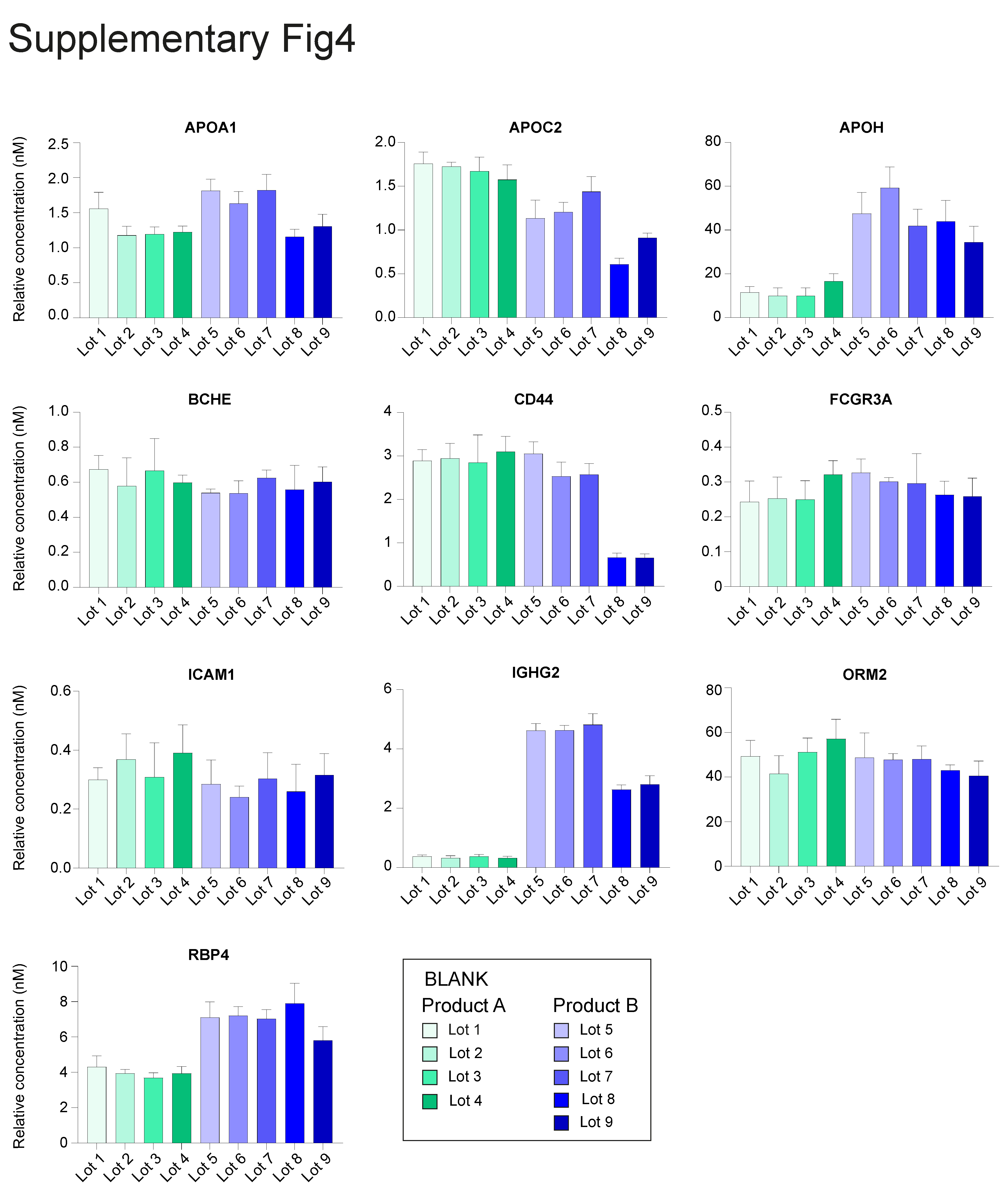

Supplement: Supplementary file 8 — Supplementary file4 (TIF 3073 KB) [file 10815_2025_3616_MOESM4_ESM.tif]

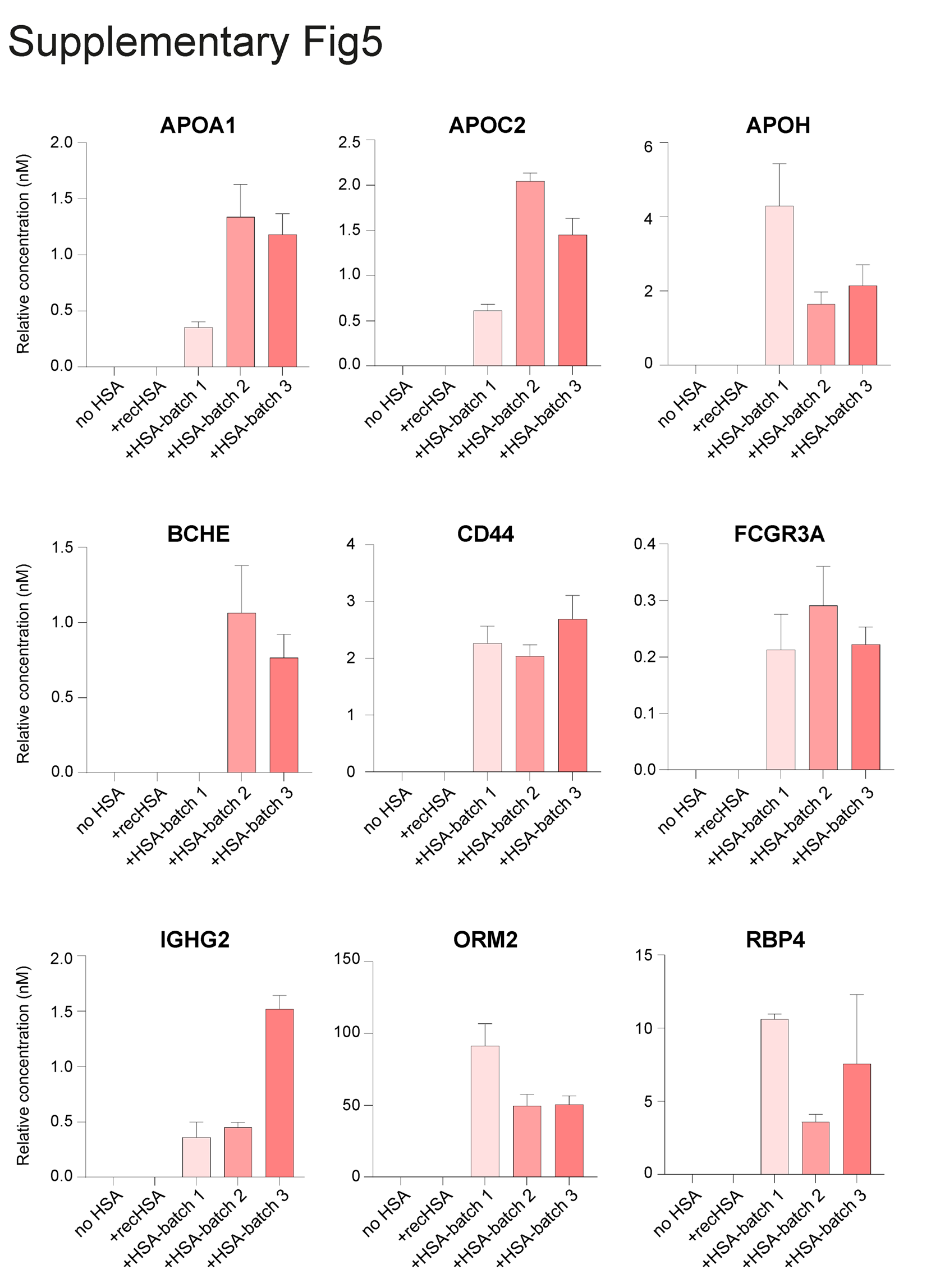

Supplement: Supplementary file 9 — PNG (324 KB) [file 10815_2025_3616_Fig11_ESM.png]

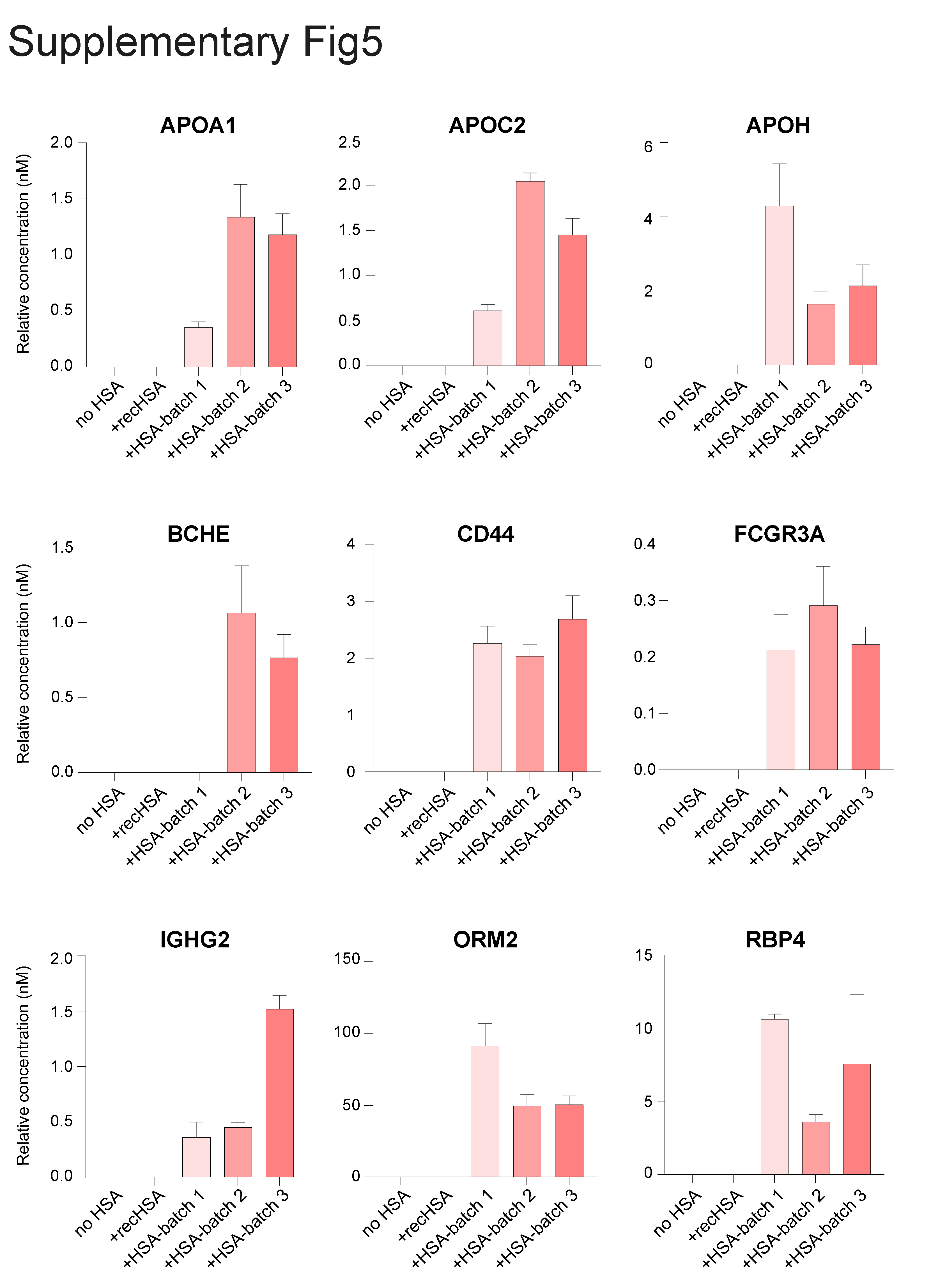

Supplement: Supplementary file 10 — Supplementary file5 (TIF 2177 KB) [file 10815_2025_3616_MOESM5_ESM.tif]

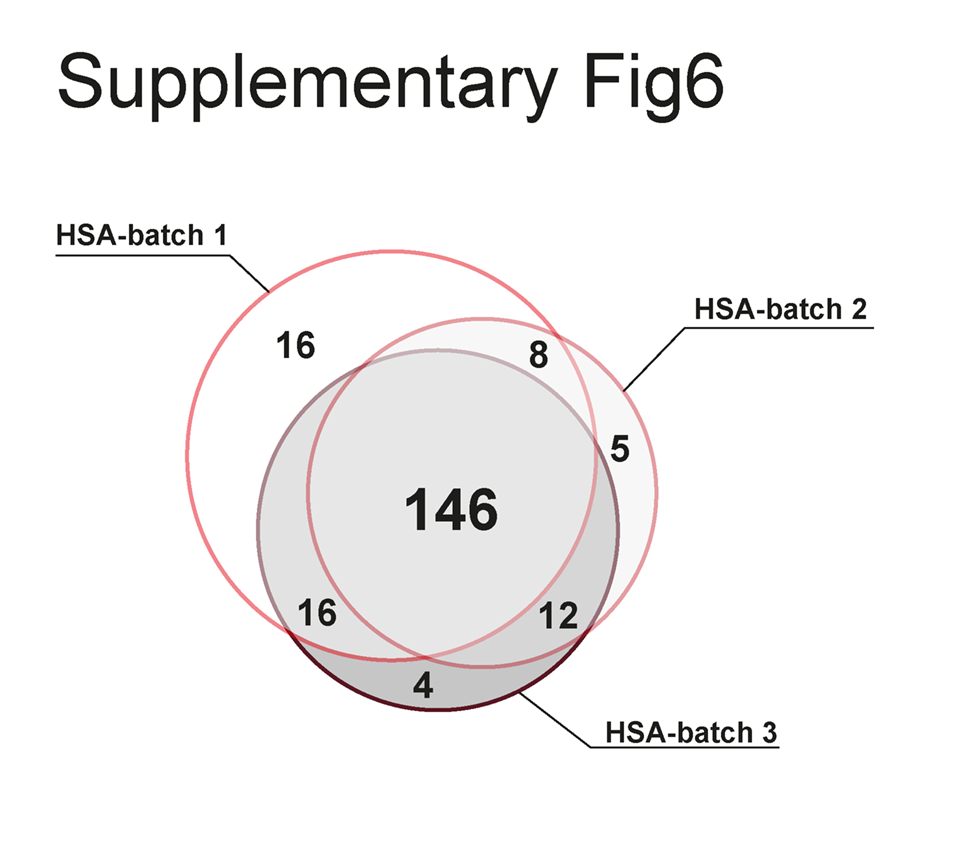

Supplement: Supplementary file 11 — PNG (101 KB) [file 10815_2025_3616_Fig12_ESM.png]

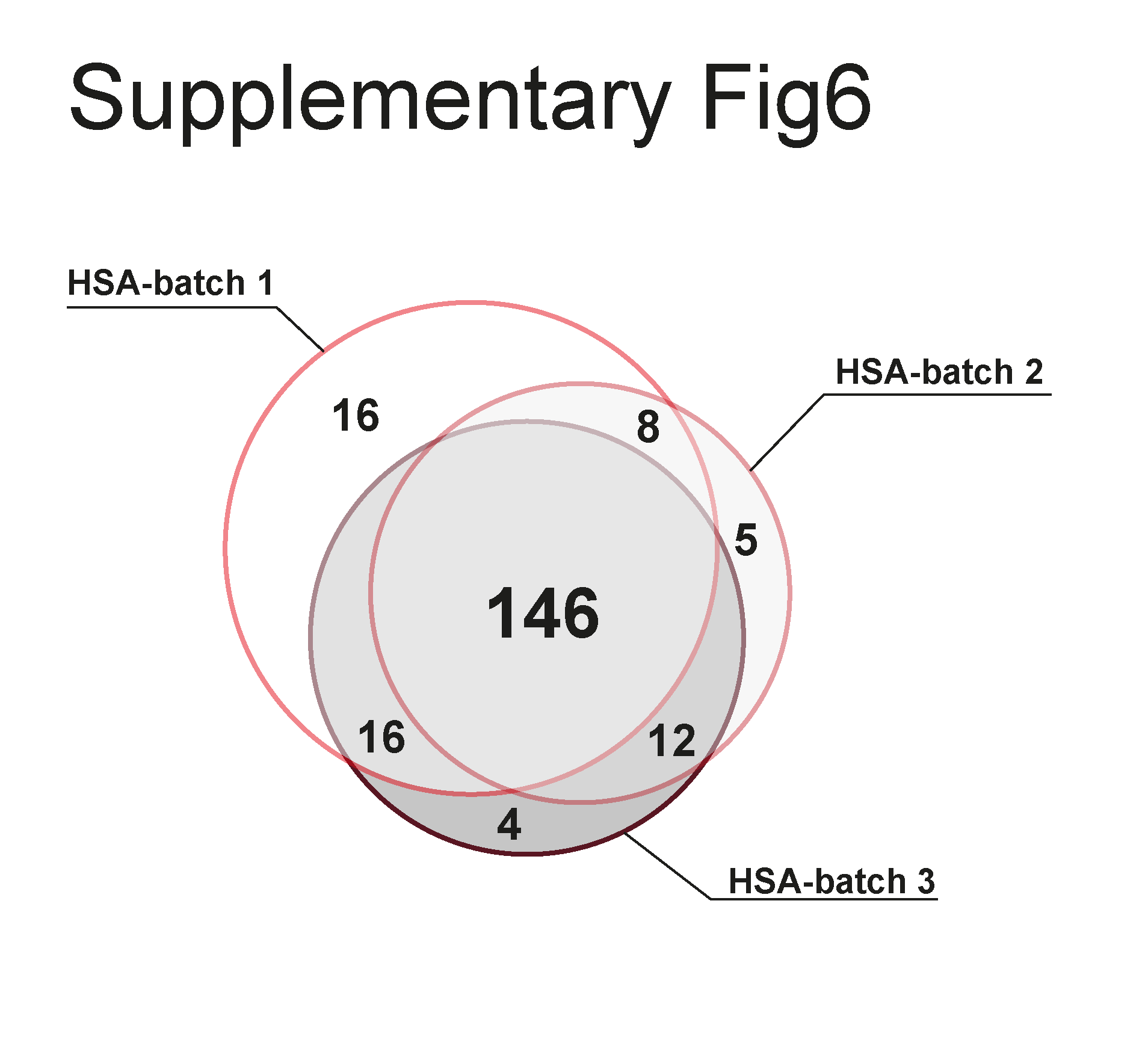

Supplement: Supplementary file 12 — Supplementary file6 (TIF 434 KB) [file 10815_2025_3616_MOESM6_ESM.tif]
