# Supplementary material for: Non-standardized protein background in IVF media linked to serum-derived albumin supplementation
Source: J Assist Reprod Genet. 2025 Sep 13;42(10):3535–49. doi: 10.1007/s10815-025-03616-0 (PMC12602788; doi:10.1007/s10815-025-03616-0)
Supplement: Supplementary file 16 — Supplementary file10 (DOCX 25.3 KB) [file 10815_2025_3616_MOESM10_ESM.docx]

**SUPPLEMENTARY MATERIAL:**

**Supplementary Fig1: List of analyzed commercial IVF culture media.** Product information and lot mapping.

**Supplementary Fig2: Additional results from protein analysis of embryo culture media.** Results for 10 additional proteins detected by targeted proteomic analysis in a subset of SCM samples (n=17) from good-quality (SPENT+, n = 8) and poor-quality (SPENT−, n = 9) embryo cultures, corresponding controls (CONTROL, n = 5), and blank samples of unused media (BLANK, n = 8 technical replicates), complementing the findings presented in **Figure 2C**.

**Supplementary Fig3: Additional results from protein analysis of spent culture media with respect to the media Lot number.** Results from targeted protein analysis of additional 10 proteins detected in SCM from good-quality (SPENT+) and poor-quality (SPENT−) embryos, categorized based on the Lot number (Lot #01-04) of the culture media used. "ns" indicates statistically non-significant differences (Mann-Whitney test, p>0.05). (n) denotes the number of samples per group. These data complement those presented in **Fig3A**.

**Supplementary Fig4: Additional results from protein analysis of blank media samples.** Results for the additional 10 proteins detected by targeted analysis in 9 Lots of embryo culture media from two different producers—Product A (shades of green) and Product B (shades of blue). These data complement those presented in **Fig4A**.

**Supplementary Fig5: Additional results from the investigation of protein background sources in embryo culture media.** Results for the additional 9 analyzed proteins detected by targeted analysis in all types of samples from the given experiment. These data complement those presented in **Fig5C**.

**Supplementary Fig6: Protein composition of IVF media supplemented with serum-derived HSA.** Untargeted analysis of protein-free media supplemented with three different batches of serum-derived HSA (batches 1–3) revealed that the majority of proteins were shared across groups (central intersection), while some protein markers were unique to individual HSA batches.

**Supplementary Table 1:** List of detected proteins and their normalized intensities, analyzed by an untargeted proteomic approach, in the SCM samples from good-quality ([+], SPENT+, n=10), poor-quality ([-], SPENT-, n=8) embryos and their corresponding controls ([C], n=12).

**Supplementary Table 2:** Proteins detected in control samples (n = 12), with mean normalized intensity values (Ctrl_mean). Each sheet lists protein species assigned to one of the following categories based on Gene Ontology classification: Cell adhesion, Cell death, DNA damage response, Growth factor activity, Hormone activity, Immune response, and Metabolism.

**Supplementary Table 3:** List of selected proteins analyzed by targeted proteomics, associated with metabolism, immune response, cell adhesion, and developmental regulation. Protein identifiers and annotated biological functions are included.
